# Food Source Identification of Macrozoobenthos in the Mangrove Ecosystem of Lubuk Damar, Aceh Tamiang, Indonesia: A Stable Isotope Approach

**DOI:** 10.21315/tlsr2024.35.2.2

**Published:** 2024-07-31

**Authors:** Ananingtyas S. Darmarini, Yusli Wardiatno, Tri Prartono, Kadarwan Soewardi, Irwan Iskandar, Musti’atin, Sonja Kleinertz

**Affiliations:** 1 Faculty of Agriculture, Djuanda University, Ciawi-Bogor, West Java 16720, Indonesia; 2 Department of Aquatic Resources Management, Faculty of Fisheries and Marine Science, Bogor Agricultural University, Bogor 16680, Indonesia; 3 Department of Marine Sciences and Technology, Faculty of Fisheries and Marine Science, Bogor Agricultural University, Bogor 16680, Indonesia; 4 Faculty of Mining and Petroleum Engineering, Bandung Institute of Technology, Bandung 40132, Indonesia; 5 Environmental Research Centre, IPB University, IPB Dramaga Campus, Bogor 16680, Indonesia; 6 Centre for Coastal and Marine Resource Studies, IPB University, Kampus IPB Baranangsiang, Bogor 16143, Indonesia; 7 Faculty of Fisheries and Marine Sciences, IPB University, Jl. Agatis Kampus IPB Dramaga, Bogor, Indonesia; 8 Marine Ecology Department, Faculty of Biology and Chemistry, University of Bremen, Loebener Str. 6, 28359 Bremen, Germany

**Keywords:** Stable Isotopes, Food Chain, Nitrogen, Invertebrates, Assimilation

## Abstract

Changes in the existence of mangroves will have an impact on changes in food webs in their respective areas. The purpose of this study was to determine the food source of the macrozoobenthos community within the Lubuk Damar mangrove ecosystem. Stable isotopes, carbon and nitrogen were used to describe the food sources for macrozoobenthos in the mangrove ecosystem of the Lubuk Damar Ecosystem, Aceh Tamiang, Indonesia. The stable isotope analysis of ^13^C and ^15^N was carried out using Isotopic-Ratio Mass Spectrometry. Potential food sources at the study site based on stable isotope ratios ranged between −29.08‰ to −20.66‰ (δ^13^C) and 4.07‰ to 5.63‰ (δ^15^N); macrozoobenthos −25.00‰ to −14.76‰ (δ^13^C) and 5.59‰ to 7.73‰ (δ^15^N). The potential food sources tested at the study site consisted of seven sources, but not all food sources in the ecosystem were consumed by the invertebrate community. This study shows that mangrove leaf litter serves as a food source for some invertebrates, such as the bivalves, gastropods, polychaetes, sipunculans, brachiopods and crustaceans. The results of this study evidence that the examined mangrove ecosystem has a function as a provider of food sources in the surrounding waters, therefore its existence is very important supporting diversity of coastal waters.

**Highlights**

- The mangrove ecosystem serves as a provider of food sources and is crucial in supporting the diversity of biota in the adjacent waters.
- The stable isotope ratios of carbon and nitrogen in macrozoobenthos range from −25.00‰ to −14.76‰ (δ^13^C) and from 5.59‰ to 7.73‰ (δ^15^N).
- Several macrozoobenthic species within the mangrove ecosystem have a direct correlation with mangrove litter as their food source.

## INTRODUCTION

Mangrove ecosystems are specialised and intricate tropical-coastal habitats that play a crucial role in global marine productivity. This productivity serves as a source of energy in aquatic food webs (Sahu & Kathiresan 2019) through the transformation of mangrove litter into detritus, supporting mangrove food webs (Husain *et al*. 2020). These ecosystems also function as nursery grounds for reef fish (El-Regal & Ibrahim 2014) and as important food and breeding grounds (Arceo-Carranza *et al*. 2021).

Mangroves display high levels of primary productivity from vegetation, algae in the roots, forest floor, phytoplankton in the water column and can receive nutrients from other sources, each of which will support fisheries productivity (Scharler 2011). Mangroves also provide ecosystem services and climate change mitigation (Mao *et al*. 2021; Ahmed *et al*. 2022). Therefore, mangrove ecosystems play a key role in the maintenance and protection of tropical and subtropical marine biodiversity and fulfil a very important function in global biogeochemical processes (Wang & Gu 2021) such as providing a sink for atmospheric nitrogen (Ray *et al*. 2014) and they are highly productive ecosystems (Banerjee *et al*. 2021). Mangroves also play a role in underground carbon storage in subtropical arid area (Torres *et al*. 2021) and support the global carbon cycle (Swangjang & Panishkan 2021). Many studies have attempted to disclose a complex link among the components within the mangrove ecosystems and between mangroves and offshore habitats leading to high needs for management and conservation purposes (Jennerjahn & Ittekkot 2002; Kristensen *et al*. 2008; Nagelkerken *et al*. 2008). Mangrove ecosystems provide food for various invertebrates, with invertebrates consuming food which consist of benthic microalgae, marine phytoplankton, particulate matter, sediment organic matter, mangrove detritus and meiofauna (Tue *et al*. 2012). Food webs formed in the mangrove ecosystem, directly and indirectly affect and contribute to organic matter particulates that are assimilated by primary consumers and transferred to higher trophic levels (Giarrizzo *et al*. 2011).

Food chains can also provide information related to functional ecology, habitat, and competition for food (Saikia 2016). Additionally, food webs can be used as good indicators of the functioning of aquatic ecosystems (Pasquaud *et al*. 2007). Another function is to provide patterns of feeding relationships between species, species interactions, community structure and energy transfer in ecosystems (Hui 2012). In the past Hyslop (1980) used the Index of Relative Importance (IRI) stomach analysis method, which became a way to identify the food source of an organism. Conventional diet studies depend on Stomach Contents Analysis (SCA). These methods are carried out by capturing, killing and dissecting several organisms (Elliott & Hemingway 2002) after taking them out of the biodiversity. However, SCA methods has its limitations, and only describes short-term food sources that have just been digested before (Zanden and Rasmussen 1996). Nowadays, one of the more accurate methods to trace food sources in an ecosystem is the analysis of stable isotopes. Stable isotope analysis is better in tracing an organism’s food source than gut content analysis due to its ability to combine spatial and temporal aspects that cannot be identified by gut content analysis alone, and it is furthermore applicable to microscopic or soft-bodied organisms as well (Bouillon *et al*. 2011). The knowledge of the content of macrozoobenthos food sources is very important because, according to Chen *et al*. (2017) macrozoobenthos is an important biota in coastal ecosystems that is the first group to utilise mangrove litter as a food source.

Stable isotope analysis has emerged as a versatile tool for answering questions in the fields of biogeochemistry, plant and animal physiology, migration patterns, niches and displacement, resource utilisation and dietary composition, trophic level estimation and food web function (Fry 2008). Specifically, the stable isotope of carbon δ^13^C can be used to determine the source of carbon in aquatic organisms (Ng *et al*. 2007), to determine the pattern of the energy dependence of coastal invertebrates and fish on allochthonous inputs (Glaz *et al*. 2012) and to identify food material and the proportion consumed and assimilated by animals (Carter *et al*. 2019). This matter provides essential knowledge that resource quality changes can increase consumer consumption at all trophic levels (Jochum *et al*. 2017).

The mangrove area in Aceh has experienced many changes since the tsunami disaster in 2004, For example, Kuta Raja, Banda Aceh has experienced shrinkage of up to 18 ha (Putra *et al*. 2016). In particular, the mangrove area of Lubuk Damar in Aceh Tamiang are areas that have been damaged, especially because previously this area had undergone land conversion. Hasri *et al*. (2014) mentioned that in 2001, the Lubuk Damar mangrove area was around 935.13 ha, and decreased in 2007 to 409.7 ha. In 2010, the area increased to 573.06 ha after replanting activities, with an average tree-level density of 230 individuals/ha.

Changes in the area and the density of mangroves are thought to have an impact on changes in the carrying capacity of mangroves as a basis for forming food webs in coastal ecosystems. Estimates of carrying capacity can be used to determine the maximum population density that can be produced under certain environmental conditions (Sarker & Wiltshire 2017). Bernardino *et al*. (2018) stated that the disappearance of the infaunal trophic diversity followed by mangrove removal suggests that large-scale forest clearing may affect estuarine food webs. The impact of mangrove damage can cause major changes in benthic ecosystem function, sediment metabolism, benthic community structure and short-term C-remineralisation dynamics for years afterward (Sweetman *et al*. 2010). The results of the study from Bernardino *et al*. (2018) revealed significant changes in the macrofaunal groups and benthic food webs in mangrove areas that have been affected by deforestation. Currently, the Lubuk Damar mangrove ecosystem consists of 12 mangrove species, 11 macrozoobenthos phyla and has at least 46 species of fishes. The research area has experienced changes in land use and illegal logging (Darmarini *et al*. 2022), which has caused the mangrove forest area to become increasingly narrow. Change in the area of mangrove will eventually change the food webs that are formed, thus highlighting the importance of this research. Data from this study, it is expected to contribute initial information about macrozoobenthos food sources from mangroves in the examined area, using a stable isotope analysis approach in the Lubuk Damar mangrove ecosystem, Seruway, Aceh Tamiang.

## MATERIALS AND METHODS

### Study Area

**Figure 1:**
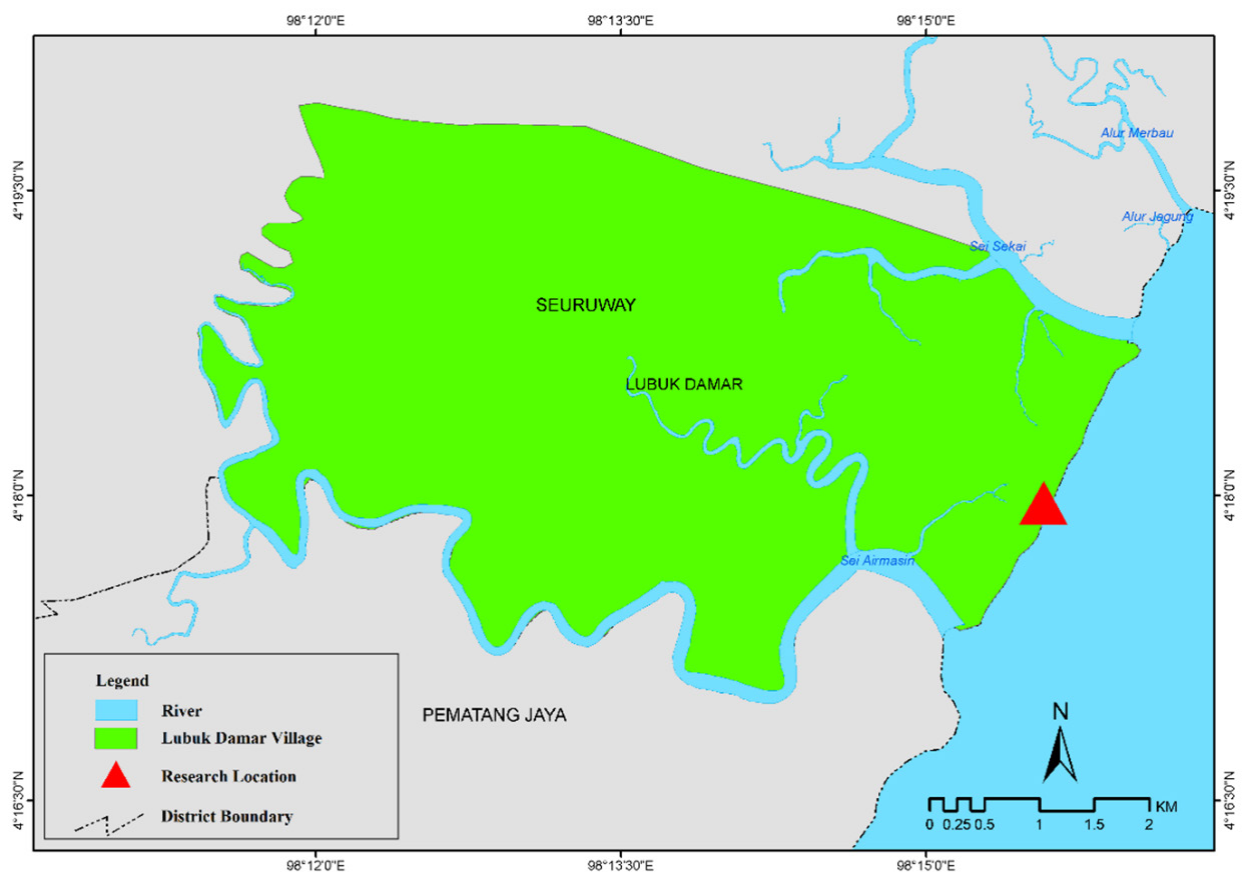
Study area in the Lubuk Damar mangrove ecosystem, Aceh Tamiang.

The research was conducted from March to July 2018 in Lubuk Damar mangrove ecosystem, Aceh Tamiang, Aceh Province. The study area was located in (98°15’44.544”E, 4°18’19.646”N) and (98°15’43.993”E, 4°18’18.131”N) to (98°15’21.138”E, 4°17’29.756”N) and 98°15’20.437”E, 4°17’28.382”N) (See Fig. 1). The Lubuk Damar mangrove ecosystem is located along the coast with a coastline length of about 3.1 km. The mangrove vegetation, in this area, comprised of approximately 12 species of mangrove trees with varying density and thickness. The shape of the sloping beach with sandy and muddy substrates has an intertidal area that is up to 1 km long at low tide.

### Sampling Technique

Substrate samples were collected using PVC cores of 5.1 cm in diameter and 20 cm in depth during low tide. The collected samples were then cleaned off of the waste. Litter samples, also collected during low tide, were taken from the uppermost substrate layer among 0 cm–5 cm, and cleaned off from the dirt and sand particles, washed with distilled water and were frozen during storage until consequent treatment. Litters, mangrove leaves and macrozoobenthos samples were obtained during the lowest tide around 0 m–200 m away from the highest tide. A total of seven samples of were analysed, i.e., substrate, litter, phytoplankton and leaves of the mangrove trees *Aegiceras floridum, Bruguiera sexangula, Excoecaria agallocha* and *Rhizophora apiculata*.

Macrozoobenthos tested consisted of annelids (*Diopatra* sp.), anthozoan, brachiopods (*Lingula* sp.), crustaceans (*Dotilla myctiroides*, *Oratosquilla* sp., *Scylla serrata*), molluscs (*Anadara* sp., *Gastrana* sp., *Pugillina* sp.) and sipunculans.

Mangrove leaves were collected by hand and placed in labelled paper envelopes. The leaves were washed to clean off the dirt (Thimdee *et al*. 2004) and cut into small pieces. Macrozoobenthos samples were taken using a PVC core diameter of 12.6 cm in size and 20 cm deep during low tide, filtered using a net of 1 mm mesh size and washed using distilled water. After washing, all substrates, litter, mangrove leaves, and macrozoobenthos samples were stored and cooled in clip plastic bags in an ice box during transportation to the laboratory to be frozen for consecutive treatments.

### Preparation of Stable Isotope Analysis

Samples were stored in a freezer until the isotope analysis period. In detail, the substrates were freeze-dried and stored in a freezer before treatment. Litter samples were dried using an oven at 60°C for 24 h and stored in a labelled bottle. Mangrove leaves were stored in freezer and freeze-dried using the freeze dryer type FDU-1200 for 2 to 5 h (Sun *et al*. 2017). In addition, macrozoobenthos samples were also freeze-dried and stored in labelled bottles.

After drying, all samples were grounded using a mortar (Jardine *et al*. 2003) and homogenised prior to the isotope test. The resulting 400 μg sample was placed in a lead tin, produced by Thermo Scientific Universal Soft Tin (OD 5 mm; H 8 mm).

### Stable Isotope Analysis

Stable δ^13^C and δ^15^N isotope analyses were conducted using Isotopic-Ratio Mass Spectrometry (IRMS) Thermo Delta V in Hydrogeology and Hydrogeochemistry Laboratory of Mining Technique Faculty, Institute Technology of Bandung, Indonesia. The isotope test used Pee Dee Belemnite (PDB) as the standard for δ^13^C and IAEA N-1 for δ^15^N. The precisions of the isotope test were 0.039‰ for δ^13^C and 0.134‰ for δ^15^N.

Isotope ratio was calculated following the method by Bouillon *et al*. (2003):


$$\delta_{X}=\frac{R_{\text{sample}}}{R_{\text{standard}}}-1\times 10^{3}‰$$

where *X* is δ^13^C or δ^15^N, and *R* represents ^13^C:^12^C ratio or ^15^N:^14^N ratio. To calculate food sources based on the value of biota assimilation the DeNiro and Epstein (1978) formula was used:


$$\Delta_{\text{Animal-diets}}=\delta^{13}{\text{C}}_{\text{animal}}-\delta^{13}{\text{C}}_{\text{diet}}$$

where Δ is signature of δ^13^C.

## RESULTS

### Stable Isotope Ratio of Food Sources and Macrozoobenthos

Stable isotope analysis on food sources in Lubuk Damar mangrove ecosystem was performed (see Table 1). Mangrove leaves originated from the species of *A. floridum, B. sexangula, E. agallocha* and *R. apiculata*. The lowest carbon isotope value was found in *B. sexangula* (−29.08‰) and the highest in litters (−20.66‰); for nitrogen isotope in *R. apiculata* (4.07‰) and the highest in litter (5.63‰). In this study the nitrogen isotope ratio of substrates was not measurable. The isotope value of stable carbon phytoplankton has similarities with *R. apiculata* with a difference of −0.75‰ and substrate with a difference of −0.03‰. Meanwhile, the nitrogen isotope value was 0.04‰ lower than in *B. sexangula*.

For the leaves of the four examined mangrove species, carbon and nitrogen isotope ratio values ranged between −29.08‰ to −26.97‰ and 4.07‰ to 5.14‰, respectively. The lowest isotope ratio of mangrove leaves was found in *B. sexangula* and the highest in *E. agallocha*. Average carbon and nitrogen isotope ratios of food sources in Lubuk Damar mangrove ecosystem were −26.79‰ and 4.03‰, respectively. Similarities of the leaves for all mangrove species in terms of carbon isotope ratios were 0.9–2.1‰ for carbon and 0.0–1.0‰ for nitrogen. The composition of stable isotope ratios of macrozoobenthos is displayed in Table 1. Carbon and nitrogen stable isotope ratios in macrozoobenthos ranged from −25.86‰ to −14.76‰ and 5.59‰ to 7.71‰. The lowest stable carbon isotope value from the tested macrozoobenthos was from the Polychaeta group (−25.00‰), while the lowest carbon isotope value was found in the Crustacea group (−14.76‰). The highest value of macrozoobenthos nitrogen stable isotope was found in Anthozoa indet. (7.73‰) and the lowest was Sipuncula indet. (5.59‰). The average value of carbon isotope stable macrozoobenthos was −19.45‰ and for nitrogen 6.53‰. The average stable carbon isotope of the tested Crustacea group was −16.81‰, while the Molluska group had a value of −18.24‰. On average the two groups above had lower carbon values than the Brachiopoda, Polychaeta and Sipuncula groups. The stable nitrogen isotope value of the crustaceans had an average value of 6.96‰, while gastropods had a value of 6.49‰.

### Food Sources of Macrozoobenthos in Mangrove Ecosystems

The tested food source compositions consisted of seven sources, i.e., phytoplankton, litter, substrates and four species of mangrove tree leaves. Not all food sources in the ecosystem were consumed by the tested invertebrate communities (Table 2). This determination is based on Bouillon *et al*. (2008) and Wardiatno *et al*. (2015) who stated that the assimilation ratio of food source carbon ranges from −2‰ to +2‰.

## DISCUSSION

Based on the carbon isotope value, *E. agallocha* has the highest value compared to other leaf types. The carbon isotope ratio of *E. agallocha* in the present study was higher than shown in Bouillon *et al*. (2003) (−28.1 *±* 0.2‰). Isotope ratios of phytoplankton in the present study were lower than displayed in Riccialdelli *et al*. (2017) (−21.0‰). Nitrogen isotope ratios in the study area were similar to phytoplankton’s nitrogen isotopes in the Bering Sea (Minagawa & Wada 1984). Litter’s carbon and nitrogen isotope ratios were −20.7‰ and 5.6‰. Substrate’s (−27.06‰) carbon isotope was lower than −17.5‰ (Bouillon *et al*. 2002a); 24.38 ± 0.9‰ (Zulkifli *et al*. 2014); −24.23‰ (Wardiatno *et al*. 2015). Substrate (−27.06‰) and phytoplankton (−27.09‰) from the study area have been shown to have the same carbon isotope ratios. The similarity of the carbon ratio between the substrate and phytoplankton is thought to be because they both have the same carbon isotope ratio.

Referring to the phylum carbon and nitrogen isotope analysis of the leaves of *A. floridum, B. sexangula, E. agallocha* and *R. apiculata* in the study area showed similar results. Carbon isotope ratios of the leaves of *A. floridum* was lower (by 1.8‰) than from *E. agallocha*. Previous studies in Segara Anakan, Java, Indonesia showed that carbon and nitrogen isotope ratios in *A. corniculatum* were −29.5 ± 0.5‰ and 4.2 ± 0.3‰, respectively (Herbon & Nordhaus 2013). Similarly, the isotope carbon ratios of *R. apiculata* and *B. sexangula* were lower 0.87‰ and 2.11‰, than from *E. agallocha*. The carbon isotope ratio of *E. agallocha* (−26.97‰) in the examined study area was similar with that of Bouillon *et al*. (2003) (−27.29‰). Carbon and nitrogen isotope ratios of *R. apiculata* were similar with those in Kristensen *et al*. (2010) and Nordhaus *et al*. (2011) (−28‰). The carbon isotope ratio of *R. apiculata* was similar to that of *R. mucronata*, only lower by 0.3‰ (Penha-Lopes *et al*. 2009). Carbon and nitrogen isotope ratios of *B. sexangula* were lower than those of *B. gymnorrhiza* in Thimdee *et al*. (2004) (−28.6‰; 4.3‰). The existence of differences in the ratio of carbon and nitrogen isotopes from the same genus but different species, that the differences in species and the area where mangroves grow can have an influence on the storage of carbon and nitrogen isotope ratios in organisms.

The average carbon and nitrogen isotope ratios of mangrove leaves were −28.2‰ and 4.4‰, respectively. The ratios were similar to those in Kenya with a difference of −0.5‰ and 0.03 ‰, respectively (Nyunja *et al*. 2009). Although on average the values are close together, the types of mangroves which constituents, differ. However, these values illustrate that mangrove carbon isotopes found in some areas tend to be low. This stems from the fact that the assimilation of young and old leaves is different according to Handagiripathira *et al*. (2015). This assumption was supported by the opinion that the ^13^C content in older leaves will be depleted due to the respiratory process in older leaves which releases CO_2_ enriched by ^13^C (Werth *et al*. 2015). Stable isotope ratios of *Anadara* sp. in the study area were −19.56‰ (δ^13^C) and 6.20‰ (δ^15^N); where the carbon isotope ratio is similar with that of *A. granosa* (−18.5‰) and lower by 3‰ than that of *A. natalensis* and the nitrogen isotope ratio is lower than that of *A. granosa* and *A. natalensis* (the processed data were taken from Bouillon *et al*. [2002b]). Stable isotope ratios of *Gastrana* sp. were similar with those of other species within the same family (Tellinidae), namely *Tellina* spp. (Bouillon *et al*. 2002b) and higher than *Tellina* sp. (−25.2‰ for δ^13^C) (Abrantes & Sheaves 2009). Carbon and nitrogen isotope ratios of *Pugillina* sp. were similar with other species within the same family (Melongenidae), i.e., *Volema cochlidium* (−18.0‰ for carbon and 9.6‰ for nitrogen) (Bouillon *et al*. 2002b). The results showed differences in species and habitat of organisms. This indicates that several organisms within the same family background display different carbon ratio equations at different locations.

Anthozoa indet. are amongst the abundant fauna detected in March 2018 and isotope ratios were −18.9‰ (δ^13^C) and 7.7‰ (δ^15^N). Dunton (2001) reported that isotope ratios of Anthozoa in Anvers Island were −24.5 ± 0.3‰ (δ^13^C) and 6.0 ± 0.1‰ (δ^15^N), and the ratios have been lower than those of Anthozoa indet. in Lubuk Damar. However, the results of the study by Nyssen *et al*. (2002) indicated that carbon isotope ratio of an Anthozoa species named *Thouarella* sp. (−16.1‰) was higher than that in Lubuk Damar (−18.86‰). Carbon and nitrogen isotope ratios of Polychaeta in general, according to Moncreiff and Sullivan (2001), were −17.7‰ and 11.6‰, respectively. Carbon and nitrogen isotope ratios of *Diopatra* sp. were lower than in average for *D. neapolitana* (Bouillon *et al*. 2002b). Different carbon isotope ratios can indicate the individuals of the same species have consumed different food sources. That different food availability may affect the distribution of animals and that this potentially shape community structure and mangrove ecosystem processes.

*D. myctiroides* is a soldier crab that is commonly found at research sites in Lubuk Damar, Aceh (Darmarini *et al*. 2019). Carbon stable isotope ratios of *D. myctiroides* were higher than those of *Scylla serrata* and similar with those of other genera within the same family, namely *Scopimera* sp. (Doi *et al*. 2005). Stable isotope ratios of *D. myctiroides* in Lubuk Damar, were lower than that of *Scopimera globusa* in Ago Bay, Japan (−10.7 ± 0.4‰ (δ^13^C) and 7.9 ± 0.7‰ (δ^15^N)) based on research of Ishishi and Yokoyama (2009). Carbon isotope ratios of *Oratosquilla* sp. in Lubuk Damar were lower than other species of mantis shrimps (*Neogonodactylus bredini*) in seagrass ecosystems and in coral rubble (deVries *et al*. 2016). Ning *et al*. (2016) stated that carbon and nitrogen isotope ratios in *O. oratoria* ranged from −18.1‰ to −16.3‰ and −13.5‰ to 10.9‰, respectively. The carbon isotope ratio of *Oratosquilla* sp. was higher than in the two different study sites mentioned above. This shows that different food sources and locations, lead to the ability of macrozoobenthos species to adapt to available food sources. Terrestrial carbon sources can also have an effect (Glaz *et al*. 2012). That is reinforced by the results of research by Sasmito *et al*. (2020), which states that the absorption and carbon cycle of mangrove ecosystems and terrestrial forests are closely related. Because at least some of the carbon lost due to erosion of terrestrial forests flows into the mangrove ecosystem.

Carbon and nitrogen isotope ratios of *S. serrata* were similar with the result of a study by Demopoulus *et al*. (2008), and lower than the results of a study by Thimdee *et al*. (2004) (−17.7 ± 0.2‰). In general, stable isotope ratios of crabs in Lubuk Damar, namely *D. myctiroides* and *S. Serrata* were lower than those of crabs from Jakarta Bay (Sudaryanto *et al*. 2012). This indicates that food sources of *D. mytiroides* and *S. Serrata* in Lubuk Damar were dominated by those with low carbon isotopes. *Lingula* sp. is a primitive group of brachiopods, and this genus can be found throughout the year. Carbon and nitrogen stable isotope ratios of *Lingula* sp. were −20.67‰ and 5.9‰, respectively, which was similar to the results of the study by Bouillon *et al*. (2002b) where the nitrogen ratio was higher than in the present study by 3.4‰. However, the ratios in the present study were higher than those of other species within the same phylum, namely *Liothyrella uva* (Dunton 2001). In the present study sipunculans were the dominating taxa in the examined study area. Stable isotope ratios of the studied specimens were −24.8‰ (δ^13^C) and 5.6‰ (δ^15^N), lower than other taxa in the same class is *Golfingia vulgaris*, with values of 5.2‰ (δ^13^C) and 2.7‰ (δ^15^N), Sokolowski *et al*. (2014).

Litter carbon assimilation ratio as a potential food source for *Anadara* sp. was 1.10‰. However, other potential food sources, such as phytoplankton, substrates, four mangrove leaves and other macrozoobenthos, have not been shown to be food sources for *Anadara* sp. This was different from the carbon isotope assimilation ratio of *A. granosa* in the mangrove Andhra Pradesh, India, that revealed potential food sources from sediments to be within the isotope signal range with values of 1.4‰ and 1.25‰ (processed data from Bouillon *et al*. 2002b). In the research area, *Anadara* sp. does not consume substrate but consumes litter, which is in line with the research results of Yurimoto *et al*. (2014) who stated that intestinal analysis of *A. granosa* from several samples contained cellulose particles, including phytoplankton and diatoms. This species is also known to have cellulolytic enzyme activity in their digestive glands, which indicates that litter is a source of food supplied from mangroves and land plants.

*Gastrana* sp. showed no proximity or enrichment on its carbon assimilation isotope ratio to a potential primary food source. The study by Sokolowski *et al*. (2014) stated that clams that belong to the same family as *Gastrana* sp., showed different values of isotops ratios and revealed that based on the carbon isotope assimilation ratio, the food was from soil organic matter. Likewise, *Pugilina* sp. was not in the range signal value of the ratio of carbon isotopes linked to potential food sources. Anthozoa indet. from the intertidal area of the Lubuk Damar mangrove ecosystem, showed the same carbon ratio as the litter carbon ratio, with a dissimilarity of only 1.8‰. This condition shows the similarity between the carbon ratio of Anthozoa indet. and its food source, namely litter. The carbon isotope assimilation ratio of *Diopatra* sp. and potential food sources revealed that the species had values similar to that of phytoplankton (2.08‰), substrate (2.06‰) and leaves of *E. agallocha* (1.96‰) indicating that species in the class Polychaeta consumed all three sources of food.

The Malacostraca group, namely *D. myctiroides* and *Oratosquilla* sp. based on stable isotope values, showed that they were not in the range of isotopic signals of potential food sources of the test. These results indicate that the species did not consume the tested food sources from the substrate, litter, phytoplankton, and mangrove leaves. Both types of species are carnivorous, so they did not consume the tested samples. This is in line with the opinion of Ning *et al*. (2016) which revealed that the food sources of *O. oratoria* consisted of 38.6% bivalves, 22.9% crabs, 16.0% copepods, 13.6% shrimps and 8.9% fish. The two species from the Malacostraca group above were different from *S. serrata* which shows that *S. serrata* has assimilated carbon isotopes that were close to the food source at the study site, namely litter. The results of the study were supported by the results of research by Mamun *et al*. (2008) who showed that this type of crab has a percentage of food intake in the form of crustaceans (44.48%), followed by molluscs (26.67%), fish tissue (15.2%), debris and substrate (10.11%), unknown (2.11%) and plant material (0.67%). So, it can be assumed that *S. serrata* in the research location consumes litter as a food source, because the study states that one of the additional food sources of *S. serrata* is plant material (0.67%) and debris (10.11%).

The results indicated that the food source of *Lingula* sp. was litter (−0.01‰). The occurrence of *Lingula* sp. in Lubuk Damar has been reported by Darmarini *et al*. (2017) and the examination of the stomach contents have shown the presence of mangrove leaf crumbs, planktonic matter and detritus (Samanta *et al*. 2015). The carbon isotope assimilation ratio of Sipuncula indet. showed that the examined specimens have not approached potential food sources (litter or substrate). Although according to Murina (1984) the Sipuncula indet. have a way of eating that tends to have the possibility of its food source coming from the substrate. The results of the analysis tend to be close to the values of substrate and *E. agallocha*, while cannot be claimed as a food source based on the assimilation ratio of carbon. Table 2 shows the assimilation value of the carbon isotope ratio as a reference for determining food sources based on the assimilation of stable isotopes. The results show that the food sources are litter, substrate, plankton, and leaves of *E. agallocha*.

The food sources examined in this study showed that the substrate litter, plankton and *E. agallocha* were consumed by some consumers. These results illustrates that the existence of mangroves in the study area is very important for the sustainability of the ecosystem. Litter, plankton, and substrates are a series of resulting products from the existence of mangroves. This shows that the presence of mangrove species in an ecosystem can also affect the potential of food sources in an area. That leads to the conclusion that the mangrove ecosystem at the study site must be maintained for the sustainability of the surrounding ecosystem.

## CONCLUSION

The results of the analysis of ^13^C and ^15^N indicates the importance of the Lubuk Damar mangrove ecosystem as a provider of food sources for macrozoobenthos. The results showed that several macrozoobenthos species had a direct relationship with mangrove litter as a food source. Based on the stable carbon isotope ratio the Polychaeta group, namely *Diopatra* sp., displayed a value which was close to the stable carbon isotope ratio of phytoplankton, substrate, and leaves of *E. agallocha*, this indicates that all three were food sources for the Polychaeta group. Other benthic organisms did not show similar values with the tested food sources, presumably, they have other food sources that have not been tested in this study. Several benthic organisms have been shown to use litter in the mangrove ecosystem as a food source. This study establishes mangroves provide a variety of different food to different benthos with various feeding and metabolic requirements. Therefore, management and conservation for the preservation of mangrove species in their ecosystem is very important in order to support mangrove areas as high biodiversity habitats.

## Figures and Tables

**Table 1:** Stable isotope ratios δ^13^C (‰) and δ^15^N (‰) of food resources and macrozoobenthos in the mangrove ecosystem in Lubuk Damar mangrove ecosystem, Aceh Tamiang.

Group	Sample	δ^13^C (‰)	δ^15^N (‰)
Producers			

Other food source	Phytoplankton	−27.09	5.11
	Litter	−20.66	5.63
	Substrate	−27.06	-
Tracheophyta	*Aegiceras floridum*	−28.80	4.14
	*Exocaria agallocha*	−26.97	4.11
	*Bruguiera sexangula*	−29.08	5.14
	*Rhizophora apiculata*	−27.84	4.07

Macrozoobenthos			

Molluska	*Anadara* sp.	−19.56	6.20
	*Gastrana* sp.	−17.29	6.26
Gastropoda	*Pugilina* sp.	−17.86	7.00
Anthozoa*	Anthozoa indet.	−18.86	7.73
Polychaeta	*Diopatra* sp.	−25.00	5.75
Crustacea	*Dotilla myctiroides*	−15.73	6.13
	*Oratosquilla* sp.	−14.76	7.03
	*Scylla serrata*	−19.94	7.71
Brachiopoda	*Lingula* sp.	−20.67	5.88
Sipuncula*	Sipuncula indet.	−24.80	5.59

*Note*:

* unidentified; indet. = indeterminate

**Table 2:** Stable isotope ratios δ^13^C (‰) and δ^15^N (‰) of macrozoobenthos in Lubuk Damar mangrove ecosystem, Aceh Tamiang.

Sample	Food sources	Δ_animal-diet_	

		0δ^13^C (‰)	δ^15^N (‰)
*Anadara* sp.	Phytoplankton	7.53	1.09
	Litter	1.10	0.57
	Substrate	7.50	-
	*Aegiceras floridum*	9.24	2.07
	*Bruguiera sexangula*	9.52	1.06
	*Exocaria agallocha*	7.41	2.09
	*Rhizophora apiculata*	8.28	2.13
*Gastrana* sp.	Phytoplankton	9.79	1.14
	Litter	3.37	0.63
	Substrate	9.77	-
	*Aegiceras floridum*	11.51	2.12
	*Bruguiera sexangula*	11.79	1.12
	*Exocaria agallocha*	9.67	2.14
	*Rhizophora apiculata*	10.55	2.18
*Pugillina* sp.	Phytoplankton	9.23	1.88
	Litter	2.80	1.37
	Substrate	9.20	-
	*Aegiceras floridum*	10.94	2.86
	*Bruguiera sexangula*	11.22	1.86
	*Exocaria agallocha*	9.11	2.88
	*Rhizophora apiculata*	9.98	2.92
Anthozoa indet.	Phytoplankton	8.23	2.61
	Litter	1.81	2.10
	Substrate	8.21	-
	*Aegiceras floridum*	9.94	3.59
	*Bruguiera sexangula*	10.23	2.59
	*Exocaria agallocha*	8.11	3.61
	*Rhizophora apiculata*	8.98	3.65
*Diopatra* sp.	Phytoplankton	2.08	0.63
	Litter	−4.34	0.12
	Substrates	2.06	-
	*Aegiceras floridum*	3.80	1.61
	*Bruguiera sexangula*	4.08	0.61
	*Exocaria agallocha*	1.96	1.64
	*Rhizophora apiculata*	2.84	1.67
*Dotilla myctiroides*	Phytoplankton	11.35	1.01
	Litter	4.93	0.50
	Substrates	11.33	-
	*Aegiceras floridum*	13.07	1.99
	*Bruguiera sexangula*	13.35	0.99
	*Exocaria agallocha*	11.24	2.02
	*Rhizophora apiculata*	12.11	2.05
*Oratosquilla* sp.	Phytoplankton	12.33	1.92
	Litter	5.91	1.41
	Substrate	12.31	-
	*Aegiceras floridum*	14.04	2.90
	*Bruguiera sexangula*	14.33	1.89
	*Excoecaria agallocha*	12.21	2.92
	*Rhizophora apiculata*	13.08	2.96
*Scylla serrata*	Phytoplankton	7.15	2.59
	Litter	0.73	2.08
	Substrate	7.13	-
	*Aegiceras floridum*	8.86	3.57
	*Bruguiera sexangula*	9.15	2.57
	*Excoecaria agallocha*	7.03	3.60
	*Rhizophora apiculata*	7.90	3.63
*Lingula* sp.	Phytoplankton	6.41	0.77
	Litter	−0.01	0.25
	Substrates	6.39	-
	*Aegiceras floridum*	8.13	1.75
	*Bruguiera sexangula*	8.41	0.74
	*Excoecaria agallocha*	6.29	1.77
	*Rhizophora apiculata*	7.17	1.81
Sipuncula indet.	Phytoplankton	2.28	0.48
	Litter	−4.14	−0.03
	Substrates	2.26	-
	*Aegiceras floridum*	4.00	1.46
	*Bruguiera sexangula*	4.28	0.45
	*Excoecaria agallocha*	2.16	1.48
	*Rhizophora apiculata*	3.04	1.52
